# Effects of Prunes on Bone Density in Humans: A Systematic Review and Meta-Analysis of Randomized Controlled Trials

**DOI:** 10.3390/nu18091338

**Published:** 2026-04-23

**Authors:** Yulia Treister-Goltzman, Roni Peleg

**Affiliations:** 1 Department of Family Medicine, Siaal Research Center for Family Practice and Primary Care, The Haim Doron Division of Community Health, Faculty of Health Sciences, Ben-Gurion University of the Negev, Beer-Sheva 84161, Israel; pelegr@bgu.ac.il; 2 Clalit Health Services, Southern District, Beer-Sheva 84161, Israel

**Keywords:** osteoporosis prevention, prunes, dried plums, meta-analysis, bone mineral density, bone biomarkers, bone resorption, bone formation

## Abstract

**Background:** Recent studies suggest that prunes are one of the most effective fruits for preventing and reversing bone loss. **Objectives:** The purpose of the present systematic review was to summarize the evidence from the randomized controlled studies on the effect of prunes on bone health in humans and to pool the results in a meta-analysis. The hypothesis of the present review was that bone mineral density of the pulled intervention group would be higher than that of the control group. **Methods:** We conducted a systematic review of randomized controlled studies with a three-level mixed-effects meta-analysis. **Results:** Of two hundred and eighty-four studies that were initially identified in PubMed, Scopus, and Web of Science using the search words, eleven papers (747 participants) were considered eligible. The effect of prune intervention in postmenopausal women was borderline significant at the lumbar spine, with BMD slightly higher in the intervention group (SMD [95% CI] = 1.30 [−0.03, 2.63]; I^2^ = 98%; *p* < 0.001). No significant differences were observed at other individual BMD sites. Heterogeneity across studies was high for all measured sites. The difference between the intervention and control groups in the levels of bone formation and resorption markers was not significant. The risk of bias of the included randomized controlled studies, assessed by the RoB v.2 tool, was low. **Conclusions:** Our meta-analysis provides preliminary evidence of modest skeletal benefits associated with consumption of 50–100 g of prunes, particularly at the lumbar spine, a trabecular-rich site. However, the overall body of research remains limited.

## 1. Introduction

Osteoporosis, the primary risk factor for fragility fractures, is highly prevalent and projected to increase as populations age globally [1,2]. Fragility fractures, particularly of the hip, spine, and wrist, are associated with substantial morbidity, loss of independence, increased mortality, and considerable healthcare costs, making osteoporosis a significant public health concern [1,2]. Effective prevention is therefore critical to reduce the clinical and economic burden of osteoporotic fractures. Diet and lifestyle factors are increasingly recognized as modulators of skeletal health, with interest growing in whole-food interventions that may support bone formation or attenuate resorption.

Prunes (*Prunus domestica*), commonly consumed as dried plums, contain a diverse array of bioactive constituents with biologically plausible roles in bone metabolism, including polyphenols, potassium, boron, vitamin K, and dietary fiber [3,4]. Commercial prunes are produced by dehydrating the cultivar *Prunus domestica* L., a variety characterized by a naturally high sugar content that allows drying while retaining the pit and without undergoing fermentation [3]. Notably, prunes rank among the highest in oxygen radical absorbance capacity of commonly consumed fruits and vegetables, suggesting potential biological effects that extend beyond basic nutritional requirements [3].

A central mechanistic hypothesis is that prune consumption may modulate inflammation and oxidative stress, two interrelated processes that play pivotal roles in bone remodeling. The phenolic compounds in prunes help scavenge reactive oxygen species and enhance endogenous antioxidant enzyme activity, thereby reducing pro-oxidant signaling that can stimulate bone resorption [4,5]. In addition, vitamin K contributes to the γ-carboxylation of osteocalcin, a process essential for proper bone matrix formation. Other prune-derived micronutrients, such as boron and potassium, have been implicated in calcium metabolism and acid-base homeostasis, providing complementary pathways through which prunes may influence skeletal integrity [3].

An emerging mechanism involves the interaction between prune consumption, the gut microbiome, and bone health [6,7]. The dietary fibers and polyphenols in prunes serve as substrates for microbial fermentation, leading to alterations in gut microbial composition and metabolic activity. These microbiome-mediated changes may generate bioactive metabolites that influence systemic inflammation, calcium absorption, and immune regulation, processes closely linked to bone remodeling. Evidence from multiple in vitro biochemical and cell culture studies [8,9,10], as well as preclinical investigations using ovariectomized and estrogen-deficient rodent models [11,12,13,14,15,16], consistently supports a favorable effect of prunes or prune-derived components on bone metabolism. Across these experimental models, prune supplementation has been shown to restore trabecular bone volume, improve bone microarchitectural parameters, and normalize biomarkers of bone resorption and formation, providing a strong foundation for evaluating the skeletal benefits of prunes.

While extensive laboratory and preclinical studies provide mechanistic support for the beneficial effects of prunes on bone, evidence from well-controlled human trials remains limited and insufficient to draw definitive conclusions. Existing randomized controlled trials (RCTs) vary in study population, intervention duration, prune dose, and skeletal outcomes, including bone mineral density and bone turnover markers. Individual trials are often small and underpowered, yielding inconsistent findings that preclude definitive conclusions. A systematic review and meta-analysis of RCTs is therefore warranted to quantitatively synthesize the evidence, improve statistical precision, and determine the overall efficacy of prune supplementation for bone health.

The purpose of the present systematic review was to summarize the evidence from the randomized controlled studies on the effect of prunes on bone health in humans and to pool the results in a meta-analysis. If found useful, people with risk-factors for osteoporosis will benefit from this simple dietary intervention.

## 2. Methods and Materials

### 2.1. Data Sources and Searches

The authors searched PubMed, Scopus, and the Web of Science electronic databases between 4 May and 30 May 2025, to identify RCTs that evaluated the effect of prunes on BMD and bone biomarkers in humans. We followed the preferred Reporting Items for Systematic reviews and Meta-Analyses (PRISMA) Reporting Guidelines for Meta-analyses. Prior to performing the review, it was registered at the PROSPERO registration site (registration # CRD420251042329).

The search was conducted using the combinations of keywords “prunes” or “dried plums” and “bone mineral density” or “bone biomarkers.” There was no limitation by date or language of publication. To identify additional studies, we reviewed the bibliographies of the full-text papers that were included in the systematic review.

Since only RCTs were included, we hypothesized that BMD at the beginning of the intervention was similar in the control and intervention groups of the studies and the mean difference in BMD at the end of the intervention was the outcome variable. This approach is accepted for the measurement of effect size in meta-analyses of studies with an experimental design [17].

### 2.2. Study Selection

The search for suitable studies was conducted in two phases according to the *a priori* inclusion and exclusion criteria. The following criteria were used for inclusion of papers into the systematic review: (1) RCTs that assessed the effect of prunes on BMD or biomarkers of bone formation/resorption in humans, (2) BMD measurements were based on Dual-energy X-ray absorptiometry and quantitative computerized tomography, and (3) the study provided the mean (±standard deviation (SD)) of BMD or biomarkers of bone formation/resorption at the end of the intervention enabling quantification of the difference between the control and the intervention groups.

The following criteria were used to exclude papers from the review: (1) not original studies, e.g., reviews, book chapters or case reports (2) interventional studies without a randomized controlled design (3) the study was conducted on a population with a specific disease (cancer, etc.).

In the first phase, all the abstracts were evaluated for the inclusion and exclusion criteria. This phase was carried out by a single investigator (YTG), due to the highly focused nature of the research question and predefined inclusion/exclusion criteria. To minimize the risk of selection bias a deliberately inclusive approach was used, whereby any study with potential relevance was retained for full-text assessment. In the second phase, both investigators read the full texts of the selected abstracts chosen in the first phase and conducted a comprehensive, independent review of all the papers in their bibliographies to identify potentially relevant papers. In cases of disagreement on a paper it was discussed until a joint decision was reached.

### 2.3. Data Extraction

Two investigators independently extracted data relevant to the study aim. All discrepancies were resolved by discussion. The data recorded included: the authors and the year of publication, the age and origin of the participating patients, the number of participants in the intervention and control groups, the site of measurement of BMD, the measured biomarker of bone resorption or formation, dosage and duration of prune administration, the results of the outcome variable (means (SD)) at the end of the intervention in both groups.

### 2.4. Quality Assessment

We used the RoB v.2 tool [18]. This tool was developed by the Cochrane Statistical Methods and Cochrane Bias Methods groups and is widely used, including in Cochrane reviews. Bias domains included in this tool are randomization, deviation from intended interventions, missing data, measurement of outcomes, and selection of reported result. The risk of bias assessment was carried out by the two investigators in a blinded process and, in cases of disagreement, a consensus process was used. Based on bias risk in all domains, the risk for bias for each study was judged as low/high/some concern.

### 2.5. Data Synthesis and Analyses

The findings of the studies were grouped according to the site of BMD measurement (lumbar spine, total hip, etc.) and the type of biomarker for bone formation/resorption. Meta-analyses were performed using the inverse-variance method with Metafor, Meta and Demtar packages for R software (version 4.3.1). As we anticipated considerable between-study heterogeneity, a random-effect model was used to pool effect sizes. Separate analyses were performed to assess the differences between the intervention and control groups for each site of bone density measurement. Subgroup analyses were performed for each biomarker. For studies that measured BMD and biomarkers several times during the intervention, only final measurement was extracted. Some studies reviewed in this meta-analysis contributed more than one observed effect (e.g., the results of interventions by different dosages), so we performed a multi-level meta-analysis to account for these dependencies: individual effect sizes (level one) which are nested within studies (level two), which compose subgroups (in case of biomarkers, level three). Standardized mean differences (SMD) with 95% confidence intervals (CIs) were calculated using Hedge’s g statistic [17] with cutoffs 0.2, 0.5, and 0.8 interpreted as small, moderate and large effects, respectively. Heterogeneity across the studies was assessed using the I^2^ (inconsistency index) measure to describe the percentage of the variability of the effect due to heterogeneity. Value above 50% or *p* < 0.1 indicated statistically significant heterogeneity.

## 3. Results

### 3.1. Study Selection

**Figure 1:**
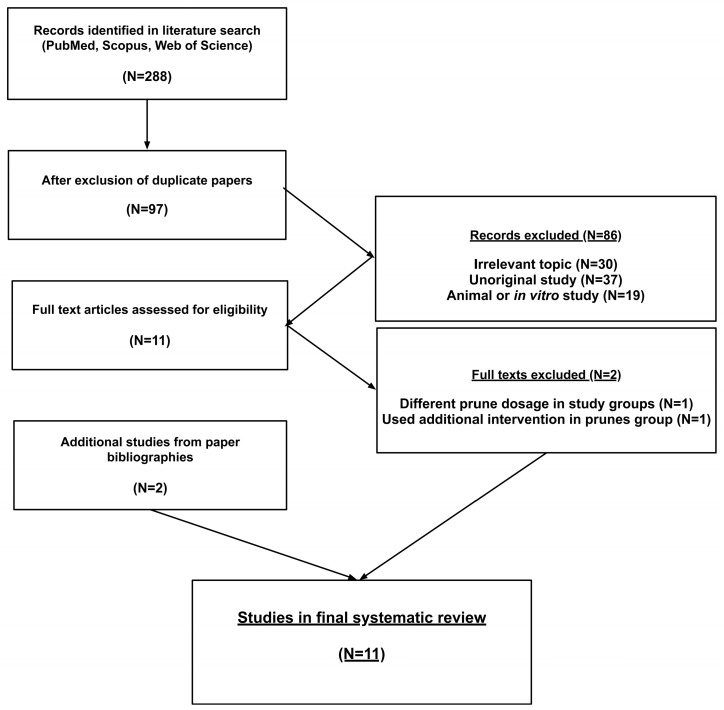
Flowchart of review process.

Two hundred and eighty-four studies were identified in PubMed, Scopus, and Web of Science, using the pre-determined keywords. Of these, 189 were duplications. In the first stage of article selection, 95 abstracts and paper titles were reviewed. Of these, 84 were excluded because it was clear from the title and/or the abstract that they did not meet the inclusion criteria. Eleven full-text papers were checked in the second stage of article selection to determine if they met the inclusion or exclusion criteria. All were published in English. One study was excluded because it compared two dosages of prunes without including a no-intervention control group [19], and another was excluded because an exercise program was implemented in the intervention group in addition to prunes [20]. A review of the bibliographies of the full papers that were included in the second phase of the review produced two additional studies that fulfilled the inclusion and exclusion criteria. Thus, at the end of the screening process, eleven papers [21,22,23,24,25,26,27,28,29,30,31] were entered into the systematic review and meta-analysis. The selection process is shown in Figure 1.

The characteristics of the articles that were included in the review are shown in Table 1. All were randomized controlled studies published in English. The year of publication ranged between 2002 and 2024. Articles [22,23,24,25,26] were based on the same population of participants, though examined different outcomes related to bone health. There was variance in sample size, from 35 patients in the study by George et al. [30] to 235 in the study, on which articles by De Souza et al. [22] and Koltun et al. [23] were based. The entire population of partcipants numbered 747 patients, 400 in the intervention group and the rest controls. While most studies were conducted among females, two studies [29,30] evaluated the effect of prunes on bone health in males. Most studies included participants older than 50 years, except for the study by DeMasi et al. [31] which included young women between the ages 18–25. In studies involving postmenopausal women, baseline BMD values were within the osteopenic range, whereas in studies including men and younger women, BMD was within the normal range. As all included studies were randomized, no significant differences in baseline BMD were observed between intervention and control groups in any study. The majority of studies were conducted in the United States, with one study [28] conducted in Korea. Seven articles [17,18,21,22,23,24,25] addressed BMD at different anatomical sites, six of which related to bone biomarkers as well [17,18,21,22,23,25]. In four articles, only bone biomarkers were addressed [15,16,19,20].

### 3.2. Outcomes: Effects of Prunes on BMD in Postmenopausal Women

**Figure 2:**
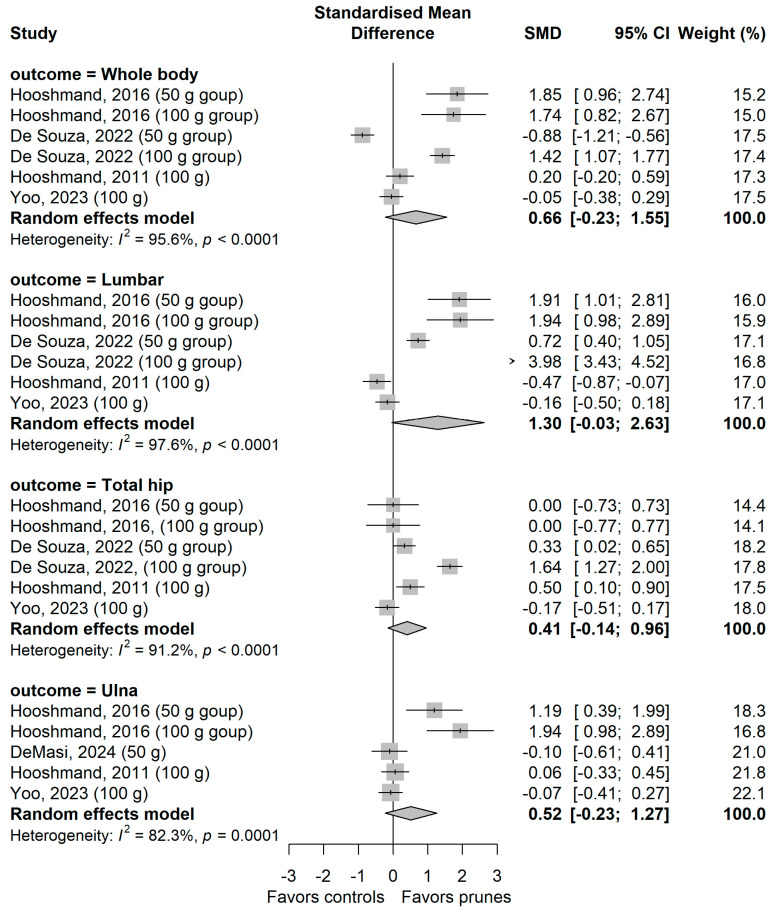
Meta-analysis on the effect of the prune intervention on bone mineral density in postmenopausal women. De Souza et al., 2022 [22]; Koltun et al., 2024 [23]; Hooshmand et al., 2011 [25]; Hooshmand et al., 2016 [27]; Yoo et al., 2023 [28]; DeMasi et al., [31].

Figure 2 shows the results of meta-analyses with pooled effects of prune intervention on different anatomical sites in postmenopausal women. The effect of intervention was borderline significant with a BMD that was slightly higher in the intervention group, at the lumbar spine site (SMD [95% CI] = 1.30 [−0.03, 2.63], I^2^ 98%, *p* < 0.001). The difference was not significant at any other of the individual BMD sites. Heterogeneity among studies was high for all other individual sites, ranging from an I^2^ of 82.3% for the ulna to 95.6% for the whole body. Sensitivity analyses, conducted by omitting one study at a time, revealed a statistically significant pooled difference between intervention and control groups for the lumbar spine when studies [25,28] were excluded (SMD [95% CI] = 1.66 [0.26, 3.06] and 1.60 [0.12, 3.08], respectively). Removing individual studies did not significantly affect results for other sites (Supplementary Figure S1).

### 3.3. Outcomes: Effects of Prunes on Biomarkers of Bone Formation and Resorption in Postmenopausal Women

**Figure 3:**
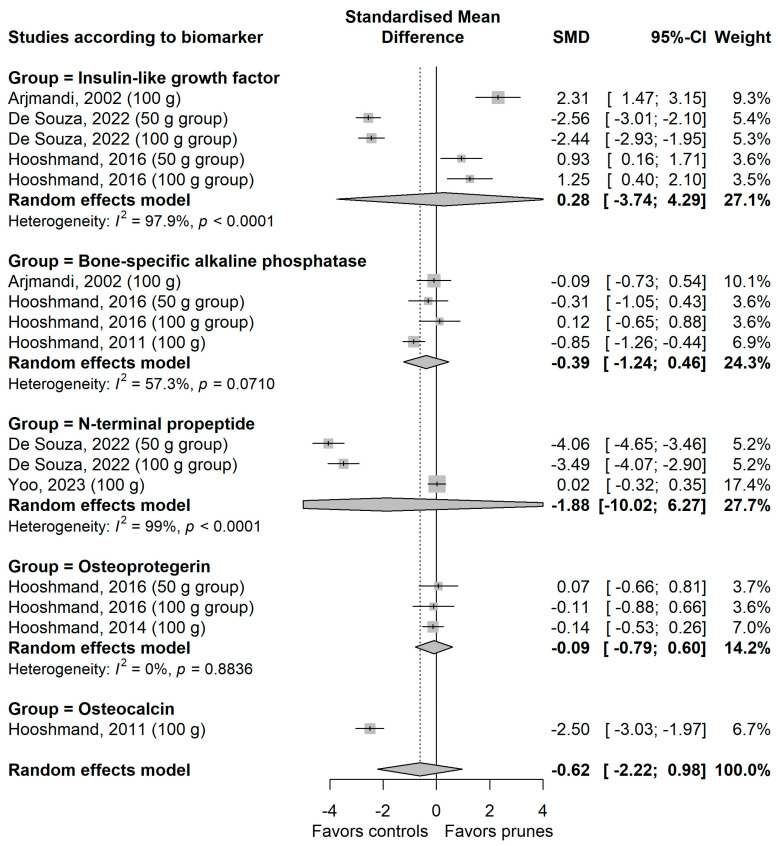
Meta-analysis on the effect of prunes on the biomarkers of bone formation in postmenopausal women. Arjmandi et al., 2002 [21]; De Souza et al., 2022 [22]; Hooshmand et al., 2011 [25]; Hooshmand et al., 2014 [26]; Hooshmand et al., 2016 [27]; Yoo et al., 2023 [28].

**Figure 4:**
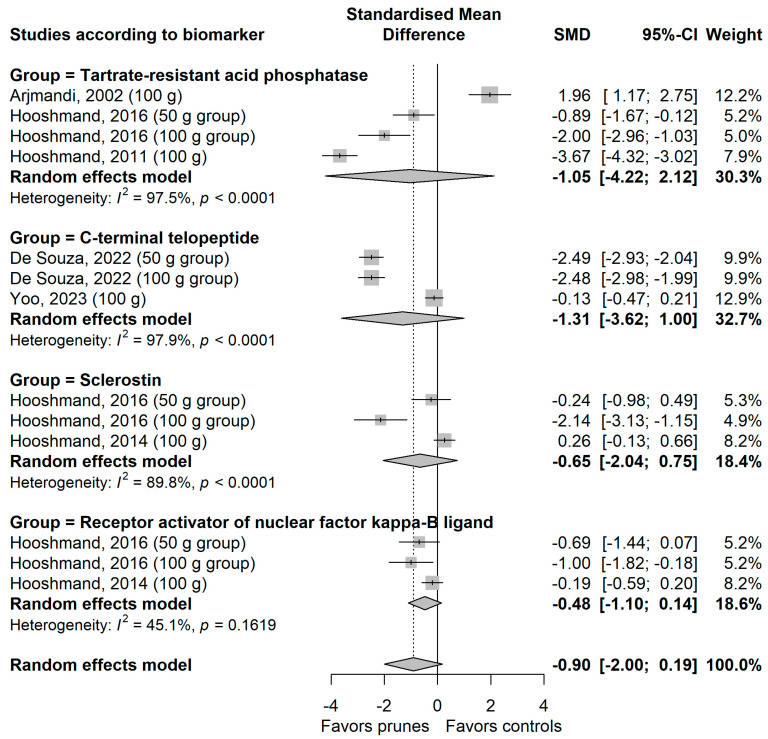
Meta-analysis on the effect of prunes on the biomarkers of bone resorption in postmenopausal women Arjmandi et al., 2002 [21]; De Souza et al., 2022 [22]; Hooshmand et al., 2011 [25]; Hooshmand et al., 2014 [26]; Hooshmand et al., 2016 [27]; Yoo et al., 2023 [28].

The meta-analysis on the effect of prune consumption on the biomarkers of bone formation is shown grouped by biomarker in Figure 3 and on the biomarkers of bone resorption in Figure 4. There were no significant differences between the prune and control groups for biomarkers of bone formation or resorption, either for the overall effect of all biomarkers (SMD [95% CI] = −0.62 [−0.22, 0.98] and −0.90 [−2.00, 0.19], respectively) or for individual biomarkers. Heterogeneity among studies was high for most biomarkers, except for receptor activator of nuclear factor-kappa b ligand and osteoprotegerin (I^2^ = 45.1% and 0.0%, respectively).

Supplementary Figure S2 shows that there was no significant pooled effect in the studies that examined the effect of prunes on CRP (SMD [95% CI] = −0.06 [−0.17, 0.29], I^2^ 0.0%, *p* = 0.564). The only study that evaluated the effects of prune consumption on cortical density and tibial strength in postmenopausal women, using quantitative computed tomography, reported an increase in BMD at the 14% diaphyseal region of the tibia, as well as improved estimated bone strength, in the pooled prune group (50 g and 100 g) compared with the control group [23]. No significant effects of prune consumption were observed on BMD at other tibial sites or on measures of bone geometry.

### 3.4. Outcomes: Effects of Prunes on BMD and Biomarkers in Populations Other than Postmenopausal Women

Three studies investigated populations other than postmenopausal women [29,30,31]. Two studies [29,30] evaluated the effects of prune consumption on bone health in men. In study [29], intake of 100 g of prunes was associated with significant reductions in bone resorption markers, tartrate-resistant acid phosphatase and C-terminal telopeptide of type I collagen, at 3, 6, and 12 months compared with baseline; however, no significant changes were observed in total body, lumbar spine, hip, or ulna BMD. Notably, endosteal circumference of the proximal tibia increased significantly within the prune group. In study [30], a 3-month intervention with 100 g of prunes significantly reduced serum osteocalcin levels, whereas consumption of 50 g of prunes decreased both osteoprotegerin and osteocalcin levels and increased the OPG:RANKL ratio compared with controls.

The only study conducted in young women compared three groups: an intervention group of oral contraceptive (OC) users and two control groups, comprising OC users and non-users [31]. The study hypothesized that prunes could potentially mitigate the impact of OC use on bone. Radius BMD increased over time in both the intervention and non-OC user control groups, whereas a reduction in trabecular density of the distal tibia was observed only in the OC user control group. It is noteworthy that sample sizes were small across all three groups, ranging from 8 to 33 participants per group.

### 3.5. Tolerance

Given the purported laxative properties of prunes, we reviewed the adverse effects reported in the included studies. In study [21], 17% of participants consuming 100 g of prunes reported gastrointestinal intolerance to the intervention. In three articles based on the same population but examining different outcomes [22,23,24], 4 of 79 participants in the 50 g prune group (5.0%) and 12 of 78 participants in the 100 g prune group (15.4%) discontinued the study due to intolerance. In two articles derived from the same sample [25,26], detailed information on discontinuation due to intolerance was not provided; however, the overall attrition rate (37.5%) was similar between the 100 g prune and control groups. In study [27], which included both 50 g and 100 g prune interventions, no participants discontinued due to prune intolerance. And in study [28], attrition was low and comparable between the 100 g prune and control groups (less than 6%), although specific reasons for withdrawal were not reported.

In one study involving males [23], 4 of 33 participants (12.1%) receiving 100 g of prunes reported gastrointestinal discomfort. In another study of males, detailed information on discontinuation due to intolerance was not provided; however, the overall attrition rate (20.0%) was similar across the 50 g prune, 100 g prune, and control groups. No intolerance to 50 g of prunes was reported in the study involving young women [31].

### 3.6. Risk of Bias of the Included Studies

As shown in Table 2, all the RCTs had a low overall risk for bias. A detailed assessment of the domains of RoB v.2 tool are presented in Supplementary Table S1. None of the studies were excluded based on quality. At the same time, it is important to note that although many of the RCTs were judged to have a low overall risk of bias, some concerns were identified in the domains of deviations from intended interventions and missing outcome data [21,22,24,25,26,27,29]. A ‘per-protocol’ analysis (excluding trial participants who did not receive their assigned intervention) and ‘as treated’ analyses (in which trial participants are grouped according to the intervention that they received, rather than according to their assigned intervention) were used in these studies. Intention to treat analyses were not performed in these studies. In accordance with methodological guidelines, dropout rates exceeding 20% are generally considered high and may increase the risk of attrition bias [32,33]. Consistent with this, attrition rates were substantial in several studies, resulting in missing outcome data that may have influenced the estimated intervention effects.

## 4. Discussion

This meta-analysis examined the effects of prunes on bone mineral density (BMD) and bone turnover markers, with most evidence derived from studies in postmenopausal women. Overall, the results suggest a potential, yet modest, impact of prune consumption on lumbar spine BMD, while no significant effects were observed at the hip, ulna, or whole body. The trend at the lumbar spine, a site rich in trabecular bone with a higher remodeling rate, may reflect the greater responsiveness of trabecular bone to metabolic and nutritional influences compared with predominantly cortical sites [34]. In addition, DXA measurements at the lumbar spine are generally more sensitive to small changes in bone density, which may further contribute to the observed differences between skeletal sites. However, the borderline nature of this effect, combined with the small number of studies and limited sample sizes, indicates that the evidence is still preliminary. It is possible that a clearer benefit could emerge in future trials with longer intervention durations and larger participant numbers. Analysis of bone turnover markers in postmenopausal women did not reveal consistent significant changes, suggesting that any skeletal effects of prunes are likely modest and may require longer exposure to become detectable. The relatively short duration of the included studies (6–12 months) further limits the ability to observe substantial changes in BMD, particularly at cortical-rich sites such as the hip and ulna, where remodeling cycles are slower [34]. The high heterogeneity observed across studies likely reflects underlying clinical and methodological variability. In the meta-analyses of bone mineral density, this heterogeneity may be primarily attributed to differences in prune dosage (50 g vs. 100 g) and intervention duration (ranging from 6 to 12 months), despite restricting analyses to specific skeletal sites. In the analyses of bone turnover markers, additional variability may arise from the inclusion of different biomarkers of bone formation and resorption, alongside differences in dosage and duration.

Taken together, the high heterogeneity, small sample sizes, and short follow-up periods limit confidence in the clinical relevance of the observed effects. Although based on conventional thresholds for SMD the observed effect sizes fall within a moderate-to-large range, the wide confidence intervals and variability across studies indicate substantial uncertainty, and it remains unclear whether this translates into meaningful clinical outcomes, such as fracture risk reduction. Three additional studies evaluated prune consumption in populations other than postmenopausal women, but the small number of studies precluded meta-analysis. Two studies in men reported reductions in bone resorption markers, and one also observed a significant increase in endosteal circumference at the proximal tibia. These findings may indicate early adaptive changes in bone structure before measurable changes in BMD. The only study in young women found an increase in ultradistal radius BMD but not at other skeletal sites, and prune intake appeared to preserve trabecular density at the distal tibia. The observed changes in BMD at the ultradistal radius and the proximal and distal tibia may be explained by the predominance of trabecular bone in these regions [34,35]. While this is physiologically plausible given the dynamic responsiveness of trabecular bone, it requires replication to confirm its significance.

Based on the present review, prunes are generally well tolerated, but higher dosages (100 g) may cause discomfort in more than 20% of patients and should be considered when interpreting the feasibility of this intervention in clinical practice.

The modest, site-specific effects observed may be attributable to the antioxidant, anti-inflammatory, and mineral-mediated properties of prunes. Reductions in bone resorption markers reported in some studies, particularly in men, support a potential attenuation of osteoclast activity through mechanisms involving oxidative stress and mineral homeostasis [36,37,38]. In contrast, inconsistent biomarker changes in postmenopausal women suggest more subtle or population-specific effects. Borderline improvements observed at trabecular-rich sites, including the lumbar spine, distal radius, and distal tibia, are consistent with their higher remodeling rates and with proposed mechanisms involving vitamin K–dependent osteocalcin carboxylation [39], polyphenols such as chlorogenic acids [40], and fiber-mediated mineral absorption [41]. Overall, prune consumption may modestly influence trabecular bone and bone remodeling dynamics rather than produce rapid increases in BMD.

Although several narrative and systematic reviews have previously examined the effects of prunes on bone health or broader health outcomes [3,4,42,43], none have quantitatively synthesized the evidence using meta-analytic methods. Earlier reviews primarily summarized preclinical findings, observational data, or a limited number of early human trials, with particular emphasis on proposed mechanisms of action and potential bone-protective effects in postmenopausal women [3,4]. Other reviews evaluated plums and prunes within a wider nutritional or health context, rather than focusing specifically on skeletal outcomes [42,43]. Importantly, none of these reviews restricted inclusion to randomized controlled trials or conducted site-specific quantitative analyses of BMD outcomes.

The present systematic review and meta-analysis extends prior work by including only randomized controlled trials in humans and by applying site-specific meta-analyses to evaluate BMD outcomes at clinically relevant skeletal sites. This approach allowed for a more rigorous assessment of the magnitude and consistency of effects across anatomical regions, while accounting for overlapping study populations and multi-arm trial designs. Furthermore, most of the included trials were published within the past three years and were therefore not captured in earlier reviews, substantially expanding the available clinical evidence base. By quantitatively synthesizing recent randomized evidence and explicitly addressing methodological dependencies and heterogeneity, this analysis provides a more critical and up-to-date evaluation of the clinical relevance of prune consumption for bone health, while also highlighting the modest nature of observed effects and the need for further adequately powered trials.

By including only high-quality RCTs and quantitatively assessing the effects of prunes on BMD, the present meta-analysis provides further support for a moderate bone-protective effect of prunes.

Limitations and strengths. The present review has several limitations. None of the included studies were blinded, due to the nature of the intervention. Additional limitations include reliance on standardized mean differences, which may reduce clinical interpretability; the potential for residual dependence among effect sizes despite the use of clustering and multi-level modeling approaches; and insufficient statistical power for analyses of individual bone sites and biomarker subgroups. Furthermore, the intervention durations in the included studies did not exceed one year, which may have limited the ability to detect potential long-term effects of prunes on bone health. Due to the small number of studies examining each bone site, dose-specific sensitivity analyses could not be conducted. Reliance on per-protocol analyses in several studies may increase the risk of attrition bias and should be considered when interpreting the findings. The observed heterogeneity, together with small sample sizes and short intervention durations, may limit the interpretability of the pooled estimates and reduce confidence in the consistency, generalizability, and clinical relevance of the findings. The present systematic review also has several strengths. It is the first to include only randomized controlled trials in humans. A systematic and comprehensive search of multiple databases was conducted to identify relevant studies, and by pooling data from multiple trials, an overall sample size of 747 participants was achieved.

## 5. Conclusions

Our meta-analysis suggests potential modest skeletal benefits of prune consumption, particularly at the lumbar spine, a trabecular-rich site. However, the overall body of research remains limited, and its clinical relevance remains uncertain. These findings should be considered exploratory in nature and interpreted with caution. Well-powered, long-term trials in diverse populations are needed to determine whether prune consumption can produce clinically meaningful effects on bone mass, turnover, or fracture risk.

## Figures and Tables

**Table 1:** Studies included in the systematic review and meta-analysis on the effect of prunes on bone health.

Study	Country and Participants	Intervention Group(s): Number of Participants; Age, Mean (SD) Years; Additional Relevant Details	Control Group(s): Number of Participants; Age, Mean (SD) Years; Additional Relevant Details	Dosage and Duration of Prunes Consumption	Outcome
a. Studies on postmenopausal women					
Arjmandi et al., 2002 [21]	USA, 38 postmenopausal women	18 women; 53.6 ± 1.6	20 women; 55 ± 1.2; received 75 g of dried apples	100 g for 3 months	IGF-1, BAP, TRAP
Studies [22,23,24] were derived from the same population of participants, the Prune Study					
De Souza et al., 2022 [22]	USA, 235 postmenopausal women	(a) 50 g intervention: 79 women; 62.0 ± 4.7 (b) 100 g intervention: 78 women; 62.3 ± 5.4; T-scores between 0.0 and −3.0, no significant differences vs. control at any site	78 women; 62.0 ± 4.8; T-scores between 0.0 and −3.0	50 g/100 g for 12 months	Total body, lumbar spine, total hip, and femoral neck BMD; CTx, P1NP, IGF-1
Koltun et al., 2024 [23]					Tibia BMD
Damani et al., 2024 [24]	USA, 183 postmenopausal women	(a) 50 g intervention: 67 women; 62.3 ± 4.6 (b) 100 g intervention: 46 women; 61.9 ± 5.5	70 women; 62.0 ± 4.8		CRP
Studies [25,26] were derived from the same population of participants					
Hooshmand et al., 2011 [25]	USA, 100 postmenopausal women 45/55 in two study groups	55 women; 57.5 ± 4.0; T-scores > −2.5, no significant differences vs. control at any site	45 women; 55.6 ± 5.0; received 75 g of dried apples; T-scores > −2.5	100 g for 12 months	Total body, lumbar spine, total hip, femoral neck, and forearm BMD; BAP, OC, TRAP, CRP
Hooshmand et al., 2014 [26]					RANKL, OPG, Sclerostin
Hooshmand et al., 2016 [27]	USA, 48 postmenopausal women	(a) 50 g intervention: 16 women; 68.5 ± 4.3 (b) 100 g intervention: 16 women; 70.4 ± 3.7; supplementation with vitamin D and calcium in both groups; T-scores > −2.5, no significant differences vs. control at any site	16 women; 71.0 ± 2.9; supplementation with vitamin D and calcium; T-scores > −2.5	50 g/100 g for 6 months	Total body, lumbar spine, total hip, forearm BMD; BAP, TRAP, CRP, IGF-1, Sclerostin, RANKL, OPG
Yoo et al., 2023 [28]	Korea, 135 postmenopausal women	72 women; 60.3 ± 6.1; supplementation with vitamin D and calcium; T-scores between −1.0 and −2.5	63 women; 61.5 ± 5.6; supplementation with vitamin D and calcium; T-scores between −1.0 and −2.5	100 g for 12 months	Lumbar spine, total hip, and forearm BMD; CTx, P1NP
b. Studies on males					
Hooshmand et al., 2022 [29]	USA, 66 males	33 males; 62.0 ± 12.9; Normal bone density, no significant differences vs. control at any site	33 males; 62.1 ± 13.1 Normal bone density	100 g for 12 months	Total body, lumbar spine, total hip, forearm, and tibia BMD; BAP, TRAP, CTx, P1NP
George et al., 2022 [30]	USA, 35 males	(a) 50 g intervention: 12 males; 69.1 ± 5.9 (b) 100 g intervention: 15 males; 65.9 ± 7.3; supplementation with vitamin D and calcium in both groups	8 males; 64.9 ± 5.9; supplementation with vitamin D and calcium	50 g/100 g for 12 months	CRP, OPG, OC, BAP, RANKL, Sclerostin, TRAP
c. Study on young women					
DeMasi et al., 2024 [31]	USA, 90 young women	30 women; 21.5 ± 1.9; OC users; Normal bone density, no significant differences vs. control at any site	(a) OC* users: 30 women; 20.9 ± 1.6 (b) Non-OC* users: 30 women; 21.4 ± 1.9; Normal bone density	50 g for 12 months	Total body, lumbar spine, total hip, femoral neck, forearm, and tibia BMD; BAP, TRAP, CRP

CTx—C-terminal telopeptide of type 1 collagen, P1NP—N-terminal propeptide of type I procollagen, IGF—insulin-like growth factor-1, CRP—C-reactive protein, OPG—osteoprotegerin, OC—osteocalcin, BAP—bone-specific alkaline phosphatase, RANKL—receptor activator of nuclear factor kappa-B ligand, TRAP—tartrate-resistant acid phosphatase, BMD—bone mineral density, OC*—oral contraceptives.

**Table 2:** Risk of bias assessment in randomized trials (RoB 2).

Study	Domain 1: Randomization	Domain 2: Deviations from the Intended Intervention	Domain 3: Missing Outcome Data	Domain 4: Measurement of the Outcome	Domain 5: Selection of the Reported Result	Overall Risk of Bias
Arjmandi et al., 2002 [21]	L	S	S	L	L	L
De Souza et al., 2022 [22]	L	S	S	L	L	L
Koltun et al., 2024 [23]	L	L	L	L	L	L
Damani et al., 2024 [24]	L	S	S	L	L	L
Hooshmand et al., 2011 [25]	L	S	S	L	L	L
Hooshmand et al., 2014 [26]	L	S	S	L	L	L
Hooshmand et al., 2016 [27]	L	S	S	L	L	L
Yoo et al., 2023 [28]	L	L	L	L	L	L
Hooshmand et al., 2022 [29]	L	S	S	L	L	L
George et al., 2022 [30]	L	L	L	L	L	L
DeMasi et al., 2024 [31]	L	L	L	L	L	L

L—low, S—some concerns.

## Data Availability

The raw data supporting the conclusions of this article will be made available by the authors on request.

## Supplementary Materials

The following supporting information can be downloaded at: https://www.mdpi.com/article/10.3390/nu18091338/s1, Figure S1: Leave-One-Out sensitivity analysis of the effect of prunes on bone mineral density in postmenopausal women. De Souza et al., 2022 [22]; Koltun et al., 2024 [23]; Hooshmand et al., 2011 [25]; Hooshmand et al., 2016 [27]; Yoo et al., 2023 [28]; DeMasi et al., 2024 [31]. Figure S2: Meta-analysis on the effect of prunes on C-reactive protein in postmenopausal women. Damani et al., 2024 [24]; Hooshmand et al., 2011 [25]; Hooshmand et al., 2016 [27]. Table S1: Detailed RoB 2 Assessment of Risk of Bias.

## Abbreviations

BMD	Bone mineral density
CIs	Confidence intervals
CRP	C-reactive protein
SMD	Standardized mean difference
