# Supplementary material for: Effects of Prunes on Bone Density in Humans: A Systematic Review and Meta-Analysis of Randomized Controlled Trials
Source: Nutrients. 2026 Apr 23;18(9):1338. doi: 10.3390/nu18091338 (PMC13164729; doi:10.3390/nu18091338)
Supplement: Supplementary file 1 [file nutrients-18-01338-s001.zip › Supplementary Table S1.pdf]

Table S1. Detailed RoB 2 Assessment of Risk of Bias.

	Study										
	Arjmandi et al., 2002 [21]	De Souza et al., 2022 [22]	Koltun et al., 2024 [23]	Damani et al., 2024 [24]	Hooshmand et al., 2011 [25]	Hooshmand et al., 2014 [26]	Hooshmand et al., 2016 [27]	Yoo et al., 2023 [28]	Hooshmand et al., 2022 [29]	George et al., 2022 [30]	DeMasi et al., 2024 [31]
Domain 1:Risk of bias arising from the randomization process											
Was the allocation sequence random?	Y	Y	Y	Y	Y	Y	Y	Y	Y	Y	Y
Was the allocation sequence concealed until participants were enrolled and assigned to interventions?	PY	Y	Y	Y	PY	PY	PY	PY	PY	Y	Y
Did baseline differences between intervention groups suggest a problem with the randomization process?	PN	N	N	N	N	N	N	N	N	N	N
**Risk-of-bias judgement**	L	L	L	L	L	L	L	L	L	L	L
Domain 2: Risk of bias due to deviations from the intended interventions (effect of adhering to intervention)											
Were participants aware of their assigned	Y	Y	Y	Y	Y	Y	Y	Y	Y	Y	Y

intervention during the trial?											
Were carers and people delivering the interventions aware of participants' assigned intervention during the trial?	NI	PY	PY	PY	NI	NI	NI	NI	NI	NI	NI
Were important non-protocol interventions balanced across intervention groups?	PY	Y	Y	Y	Y	Y	Y	Y	Y	Y	Y
Were there failures in implementing the intervention that could have affected the outcome?	N	N	N	N	N	N	N	N	N	N	N
Was there non-adherence to the assigned intervention regimen that could have	PY	PY	PY	PY	PY	PY	PY	N	PY	N	N

affected participants' outcomes?											
Was an appropriate analysis used to estimate the effect of adhering to the intervention?	N	N	Y	N	N	N	N	Y	N	N	N
**Risk-of-bias judgement**	**S**	S	L	S	S	S	S	L	S	L	L
Domain3: Risk of bias due to missing outcome data											
Were data for this outcome available for all, or nearly all, participants randomized?	PN	PY	PY	N	N	N	N	Y	N	Y	Y
Is there evidence that the result was not biased by missing outcome data?	PN	PN	PY	N	N	N	N	PN	N	PN	PN
Could missingness in the outcome depend on its true value?	PN	PN	PN	PN	PN	PN	PN	PN	PN	PN	PN
Is it	PN	PN	PN	PN	PN	PN	PN	PN	PN	PN	PN

likely that missingness in the outcome depended on its true value?											
**Risk-of-bias judgement**	S	S	L	S	S	S	S	L	S	L	L
Domain 4: Risk of bias in measurement of the outcome											
Was the method of measuring the outcome inappropriate?	Y	Y	Y	Y	Y	Y	Y	Y	Y	Y	Y
Could measurement or ascertainment of the outcome have differed between intervention groups?	N	N	N	N	Y	Y	Y	Y	Y	Y	Y
Were outcome assessors aware of the intervention received by study participants?	NI	PN	PN	PN	Y	Y	Y	Y	Y	Y	Y
Could assessment of the outcome have been influenced by knowledge	N	N	N	N	Y	Y	Y	Y	Y	Y	Y

of intervention received?											
Is it likely that assessment of the outcome was influenced by knowledge of intervention received?	N	N	N	N	Y	Y	Y	Y	Y	Y	Y
**Risk-of-bias judgement**	L	L	L	L	L	L	L	L	L	L	L
Domain 5: Risk of bias in selection of the reported result											
Were the data that produced this result analysed in accordance with a pre-specified analysis plan ?	Y	Y	Y	Y	Y	Y	Y	Y	Y	Y	Y
Is the numerical result being assessed likely to have been selected, on the basis of the results, from multiple eligible	N	N	N	N	N	N	N	N	N	N	N

outcome measurements											
Is the numerical result being assessed likely to have been selected, on the basis of the results, from multiple eligible analyses of the data?	N	N	N	N	N	N	N	N	N	N	N
**Risk-of-bias judgement**	L	L	L	L	L	L	L	L	L	L	L
