# Supplementary figures and images for: Effects of Prunes on Bone Density in Humans: A Systematic Review and Meta-Analysis of Randomized Controlled Trials
Source: Nutrients. 2026 Apr 23;18(9):1338. doi: 10.3390/nu18091338 (PMC13164729; doi:10.3390/nu18091338)

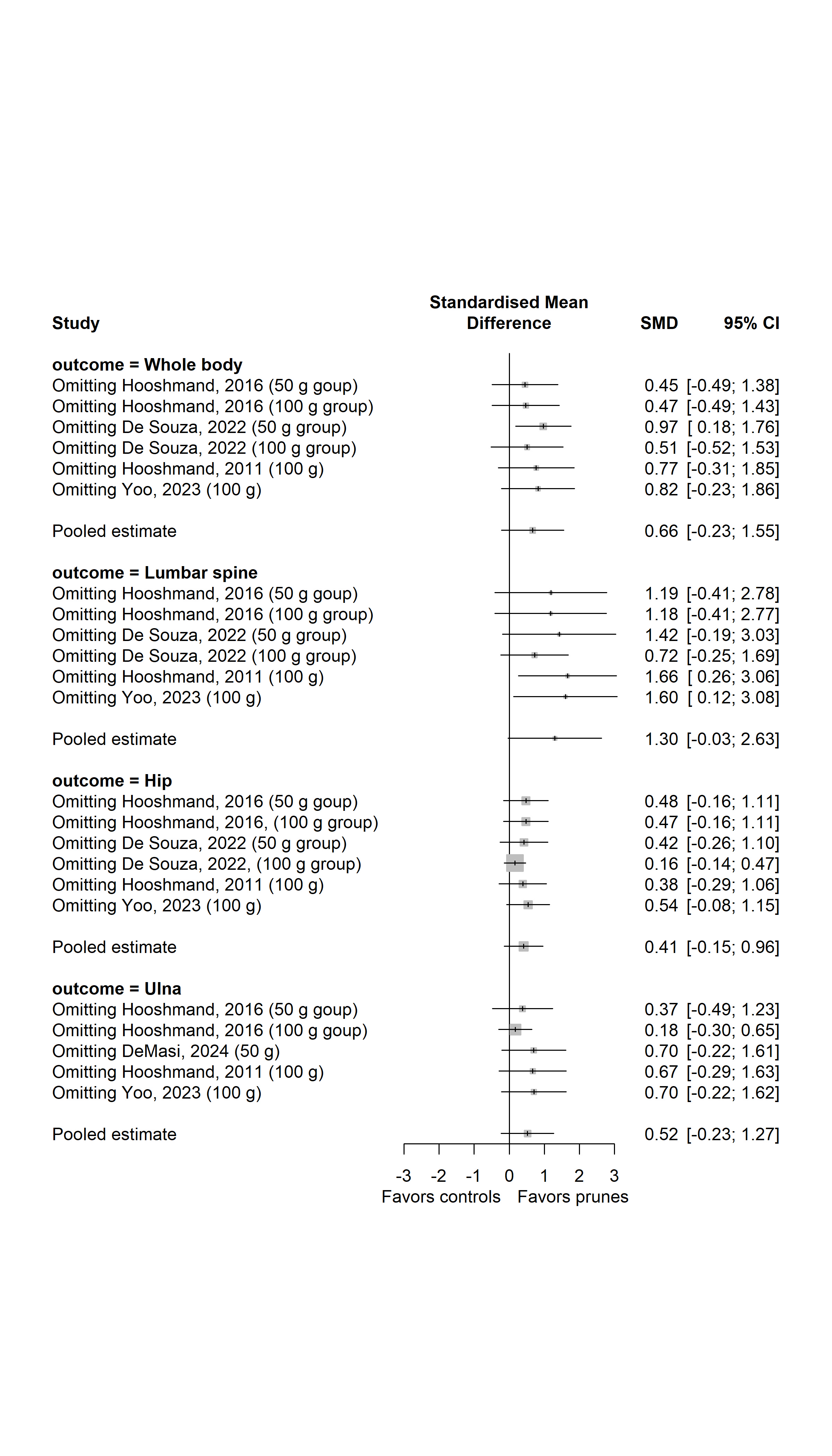

Supplement: Supplementary file 1 [file nutrients-18-01338-s001.zip › Supplementary Figure S1.tiff]

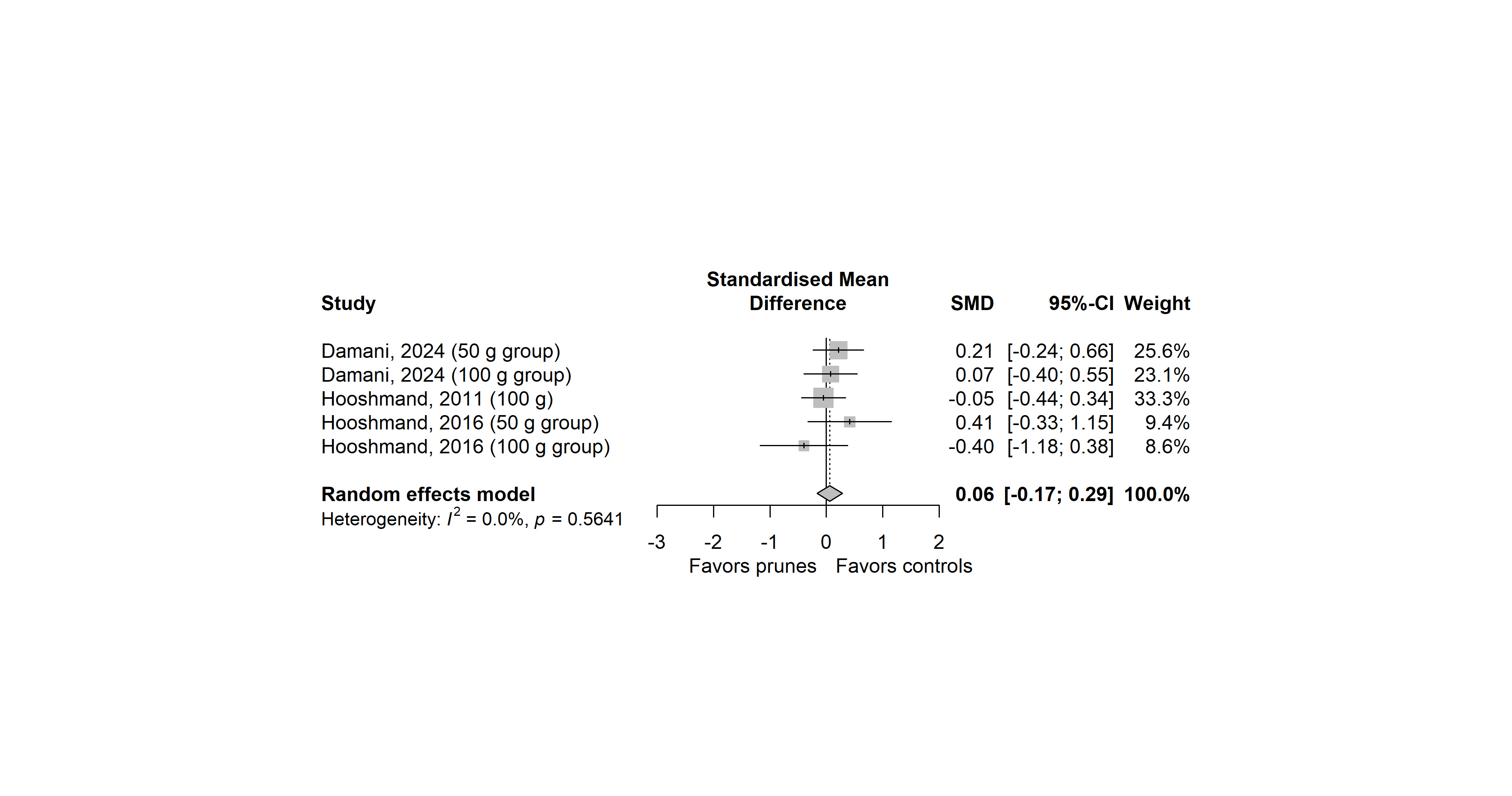

Supplement: Supplementary file 1 [file nutrients-18-01338-s001.zip › Supplementary Figure S2.tiff]
